# Fetal origin of bronchopulmonary dysplasia: contribution of intrauterine inflammation

**DOI:** 10.1186/s10020-024-00909-5

**Published:** 2024-09-03

**Authors:** Haoting Yu, Danni Li, Xinyi Zhao, Jianhua Fu

**Affiliations:** Department of Pediatrics, Shengjing Hospital of China Medical University, Shenyang, Liaoning, 110004 China

**Keywords:** Bronchopulmonary dysplasia, Intrauterine inflammation, Fetal inflammatory response, Chorioamnionitis, Alveolarization, Vascular development

## Abstract

Bronchopulmonary dysplasia (BPD) is a common chronic lung disease in infants and the most frequent adverse outcome of premature birth, despite major efforts to minimize injury. It is thought to result from aberrant repair response triggered by either prenatal or recurrent postnatal injury to the lungs during development. Intrauterine inflammation is an important risk factor for prenatal lung injury, which is also increasingly linked to BPD. However, the specific mechanisms remain unclear. This review summarizes clinical and animal research linking intrauterine inflammation to BPD. We assess how intrauterine inflammation affects lung alveolarization and vascular development. In addition, we discuss prenatal therapeutic strategies targeting intrauterine inflammation to prevent or treat BPD.

## Introduction

Bronchopulmonary dysplasia (BPD) is a multifactorial chronic lung disease and is a leading cause of morbidity and mortality in premature infants, particularly those with lower gestational age and birth weights (Jensen et al. 2021; Lui et al. 2019; Thébaud et al. 2019). During the past two to three decades, the incidence of BPD in preterm infants ranged from 11 to 50% in different countries, becoming increasingly prevalent because of the improved survival rates of extreme preterm births (Thébaud et al. 2019). BPD is characterized by respiratory distress and requires postnatal respiratory support, including mechanical ventilation (MV) and oxygen supplementation. Long-term sequelae of BPD, include neurodevelopmental and cognitive changes (Cheong et al. 2018), impaired lung function (Pérez-Tarazona et al. 2021), asthma (Sun et al. 2023), pulmonary vascular disease (Thébaud et al. 2019), cardiac dysfunction (Thébaud et al. 2019), and impaired immune development, causing recurrent infections and rehospitalizations (Duijts et al. 2020). “Classic” BPD first described by Northway et al. (1967), is characterized by fibrotic, scarred, and hyper-inflated lungs following MV and oxygen toxicity (Schmidt and Ramamoorthy 2022). It was initially described as a pulmonary disease affecting preterm infants with an oxygen requirement at 28 days of life (Day and Ryan 2017). Due to advancements in respiratory care, improved respiratory support techniques, and the introduction of surfactant therapy over the past fifty years, “classic” BPD has become less common (Allen and Panitch 2021). The term “new” BPD has been proposed recently and is characterized by impaired lung alveolarization and dysregulated vascularization. Specifically, it refers to reduced quantity and increased size of alveoli, structural simplicity and pulmonary microvascular dysplasia (Bancalari and Jain 2019).

BPD is affected by different prenatal and postnatal factors, including intrauterine inflammation, premature delivery, low birth weight, etc. Prenatal risk factors are strongly associated with the occurrence of BPD, regardless of advances in postnatal care (Kramer et al. 2009; Taglauer et al. 2018). BPD is typically initiated during pregnancy. Among the many factors contributing to BPD, inflammation plays a significant role in BPD onset, development, and severity (Higgins et al. 2018; Shahzad et al. 2016). Intrauterine inflammation can lead to rupture and premature delivery, which is the primary cause of BPD (Hwang and Rehan 2018). Intrauterine inflammation can also cause persistent lung inflammation in neonates, leading to developmental damage to the alveoli and pulmonary blood vessels (Dankhara et al. 2023; Salimi et al. 2022). The damage positively correlates with the severity of inflammation in the lungs and systemic organs (Choi et al. 2016). Intrauterine inflammation affects lung development through a variety of pathophysiological mechanisms. Clarifying prenatal intrauterine inflammation mechanisms is necessary to ensure the effective prevention and treatment of BPD. Therefore, we review recent evidence linking BPD development to intrauterine inflammation to investigate the fetal origin of BPD.

### Methodology

A thorough search of PubMed’s literature was conducted to find articles related to bronchopulmonary dysplasia, intrauterine inflammation, prenatal inflammation, intrauterine infection, chorioamnionitis, fetal inflammatory response and other similar terms. Information from systematic reviews was prioritized while assessing the collected literature. We carried out a narrative review of the scientific literature, building on advances in laboratory science and clinical studies to present a current assessment of the contributions made by intrauterine inflammation to pathogenesis of BPD.

## Exposure to intrauterine inflammation: definitions

Fetal intrauterine inflammation is commonly linked to chorioamnionitis, which is characterized by acutely inflamed placental membranes and chorions, usually caused by ascending microbial pathogens (Brosius Lutz et al. 2021). Histological changes include maternal chorioamnionitis, fetal funisitis, and chorionic vasculitis (Kim et al. 2015a; Muraskas et al. 2020). Microbial and inflammatory products in the amniotic fluid and choriodecidua activate the amnion and chorion, leading to neutrophil chemoattractant (CXCL-8/IL-8 and CSF3) release in an IL-1 and TNF-dependent manner (Cappelletti et al. 2020). The intra-amniotic inflammatory response, coordinated by chorion and amnion cells, fetal skin cells, and the umbilical cord, is mediated by toll-like receptor activation. This leads to pathogen-associated molecular patterns by the secretion of pro-inflammatory cytokines (Brosius Lutz et al. 2021). Chorioamnionitis is characterized by neutrophilic infiltration and inflammation at the maternal-fetal interface. There, it amplifies inflammation through interaction with other immune and resident cells (Cappelletti et al. 2020).

**Fig. 1:**
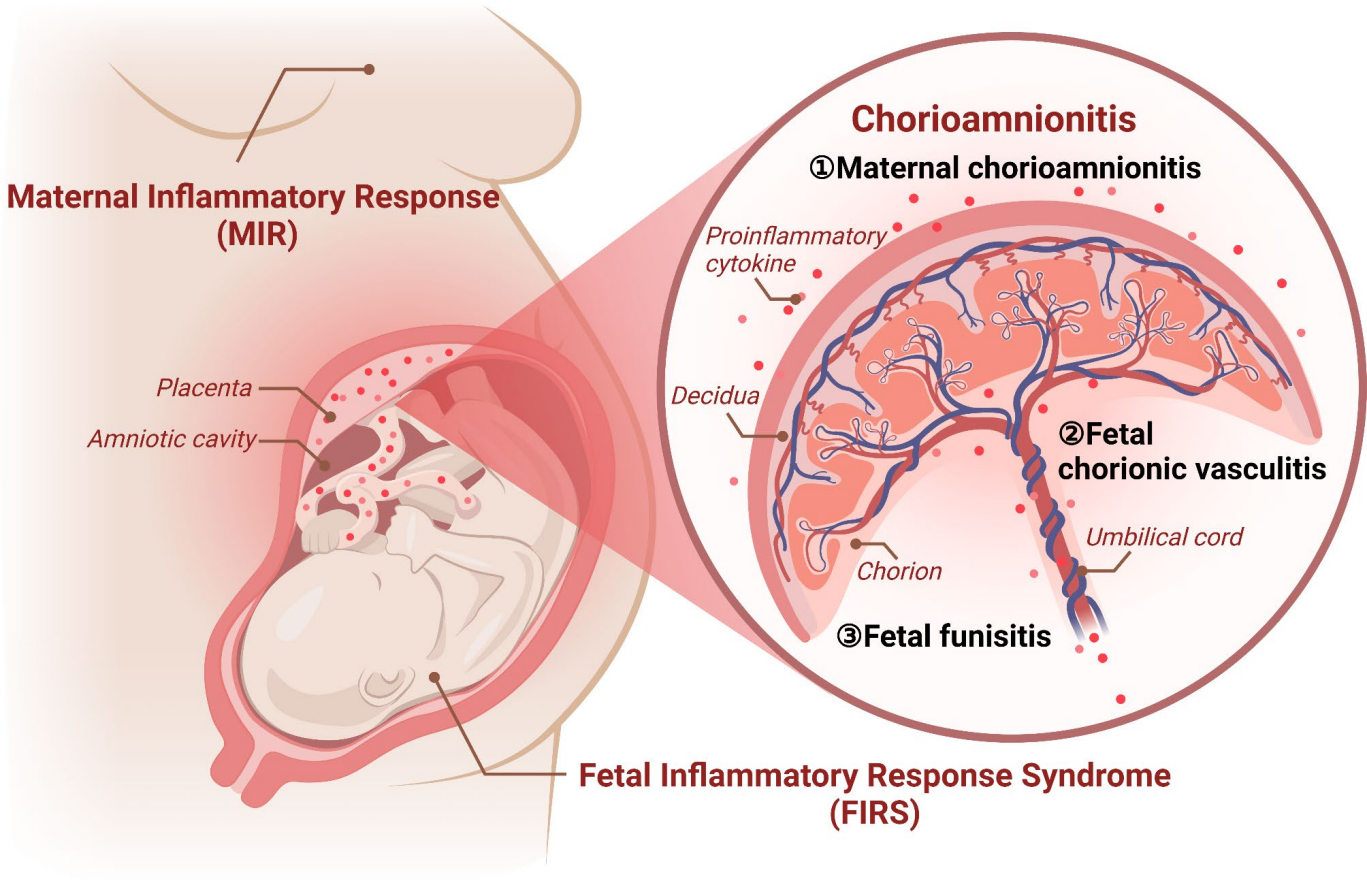
Chorioamnionitis and its relationships with maternal and fetal inflammatory response in intrauterine inflammation. The histology changes of chorioamnionitis include maternal chorioamnionitis, fetal funisitis and chorionic vasculitis. The infection invades the decidua and the amniotic cavity, leading to MIR and FIRS. Created with Biorender. com

Chorioamnionitis often occurs in the placenta of premature infants, triggering an inflammatory cascade termed the maternal inflammatory response (MIR). Excessive neutrophil production of proteases, reactive oxygen species, and inflammatory cytokines during pathogenic clearance can accelerate fetal membrane aging, leading to rupture and preterm labor (Jain et al. 2022; Zhang et al. 2023a). Chorioamnionitis affects 36% of pregnancies shorter than 32 weeks and carries a substantial risk of extremely preterm birth (Dessardo et al. 2012). When chorioamnionitis spreads into the decidua and amniotic cavity, neutrophils infiltrate the chorioamnion and the levels of pro-inflammatory cytokines increase. Maternal neutrophils migrate from decidual vessels to fetal membranes, and fetal neutrophils migrate from fetal umbilical vessels to surrounding umbilical cord tissue in response to a cytokine gradient, constituting funisitis (Brosius Lutz et al. 2021). Funisitis and chorionic vasculitis, which are symptoms of fetal inflammatory response syndrome (FIRS), are caused by immune cells that invade the blood vessels and umbilical cord as inflammation intensifies (Martinelli et al. 2012; Murthy and Kennea 2007). Figure 1 provides a detailed illustration of several definitions around intrauterine inflammation as well as the relationship between maternal and fetal inflammatory responses.

FIRS, firstly characterized by Gomez et al. (1998), as a systemic inflammatory response with elevated fetal plasma interleukin 6 (IL-6) levels (> 11 pg/mL), is a subclinical condition characterized by fetal immune system activation. In 2002, Pacora et al. (2002) proposed funisitis and chorionic vasculitis as histological manifestations of FIRS, indicative of acute histological inflammation in the placenta, extraplacental membranes, and umbilical cords. 63.5% of the preterm infants have chorioamnionitis with FIR (Kovács et al. 2023). 8% of preterm newborns up to 33 weeks of age exhibit FIRS without MIR/FIR, accounting for 29% of all FIRS cases. Nomiyama et al. (2023) recommended testing IL-6 levels in umbilical cord blood to prevent missed FIRS diagnosis, and development of BPD and other morbidities. This applies to premature infants born to mothers with low risk of intra-amniotic inflammation (Nomiyama et al. 2023).

## Relationship between intrauterine inflammation and BPD: recent evidence

### Clinical evidence

Exposure to chorioamnionitis increases the risk of developing BPD in preterm infants, but does not influence the grade of BPD. A meta-analysis of 59 studies (1994–2009) involving 15,295 preterm infants found that chorioamnionitis exposure nearly doubled the odds of developing BPD (Hartling et al. 2012). Villamor-Martinez et al. (2019) presented an updated systematic review in 2019 that included 158 studies, 244,096 infants, and a wider range of covariates. They found that chorioamnionitis was a significant risk factor for BPD in preterm infants, regardless of the definition criteria for BPD. The impact of chorioamnionitis on BPD in their study may be modulated by its effect on gestational age and the risk of respiratory distress syndrome, but a significant link with BPD severity was not established (Villamor-Martinez et al. 2019).

The histological location and severity of intrauterine inflammation may influence its association with BPD (Kim et al. 2015b). Several studies have shown that BPD is more closely related to chorioamnionitis than to funisitis, suggesting lung inflammation directly contacting the airway epithelium may have a greater predisposition to BPD than systemic inflammation through the umbilical cord during the fetal period (Kim et al. 2015b; Villamor-Martinez et al. 2019). Histologically, both MIR and FIR can be classified into three stages and two grades, with necrotizing chorioamnionitis and necrotizing funisitis as the most severe stages respectively (Khong, et al. 2016). The incidence of BPD increased with the increased stage (or grade) of chorioamnionitis or funisitis (Costa, et al. 2023; Lee, et al. 2015b). This upward trend was thought to be related to earlier gestational age at delivery (Lee, et al. 2015b). Recently, Costa, et al. (2023) demonstrated that among extremely low gestational age neonates, exposure to stage 2–3 and grade 2 chorioamnionitis is an independent risk factor for the occurrence and severe form of BPD.

BPD arises from a complex interplay of pre-and/or postnatal factors, with adverse postnatal events complicating the association between intrauterine inflammation and BPD (Davidson and Berkelhamer 2017). For example, chorioamnionitis causes postnatal lung inflammation and subsequent lung injury in intubated infants, contributing to the development of BPD (Perniciaro et al. 2020). Moreover, preterm infants with respiratory distress syndrome and severe chorioamnionitis have an impaired surfactant response, which requires higher oxygen requirement and prolonged time to extubation after surfactant therapy, increasing BPD risk (Been et al. 2010).

FIRS is a risk factor for serious morbidity or mortality in newborns and is associated with an increased likelihood of unfavorable outcomes including BPD (Jung et al. 2020; Tang et al. 2021). Approximately 41.6% of newborns with gestational ages between 26 and 34 weeks exhibit FIRS (Francis et al. 2019). A systematic review and meta-analysis encompassing 10 articles and 1116 patients has shown that FIRS is linked to a higher frequency of BPD compared to neonates without FIRS (Tang et al. 2021). Furthermore, FIRS without MIR/FIR occurred in 8% of preterm births up to 33 weeks, accounting for 29% of FIRS cases. Recent study confirmed that neonates with FIRS but without MIR/FIR had the same elevated risk of BPD as neonates with FIRS and MIR/FIR (Nomiyama et al. 2023). Additionally, studies on FIR and BPD relationship indicate a significant increase in BPD odds among neonates with concurrent FIR, regardless of FIRS presence (Kovács et al. 2023).

### Animal model evidence

Recent animal model-based studies have confirmed the link between intrauterine inflammation and BPD (Table 1). These models show that the induction of intrauterine inflammation using lipopolysaccharides (LPS) or endotoxin (ETX) during critical developmental phases of the lung may variably influence lung development in experimental animals (Abele et al. 2022; Cao et al. 2009; Mandell et al. 2021; Li et al. 2016; Pan et al. 2023; Tang et al. 2010; Ueda et al. 2006; Willems et al. 2017; Zhou et al. 2022). Rat alveolarization, occurring postnatally between days 4 and 21, mimics those of premature infants (O’Reilly and Thébaud 2014). Studies typically use rats exposed to inflammation during the lung development stage that resembles the human pseudoglandular stage (24–26 weeks of gestation) to model intrauterine infections (Mandell et al. 2021). Pregnant rats received intraperitoneal, intra-amniotic, or endocervical intramuscular injections of LPS (Cao et al. 2009), ETX (Abele et al. 2022), or *Escherichia coli* (*E. coli*) (Pan et al. 2018, 2023) to model prenatal inflammation. This leads to fetal rats with persistent aberrant development of alveoli and blood vessels throughout the infant period.

**Table 1:** Animal model evidences of the relations between intrauterine inflammation and BPD

Animal model	Agent for Intrauterine Inflammation	Time of induction	Time of pulmonary evaluation	Effects of intrauterine inflammation on lung	References
WKAH/htm rats	1 µg/sac LPS	22.5 dGA (term: 22.5 dGA)	PN 3, 14, 60	Fewer and larger alveoli	Ueda et al. 2006
Wistar rats	2.5 mg/kg LPS	20, 21 dGA (term: 22 dGA)	PN 2–14	Lung morphologically immature with fewer secondary septae and fewer and larger alveoli, reduced the rate of alveolarization	Cao et al. 2009
Sprague-Dawley rats	10 µg/sac ETX diluted in 50 µL NS	20 dGA (term: 22 dGA)	PN 7, 14	Marked reduction in alveolarization and vascular growth, led to altered lung structure and pulmonary hypertension	Tang et al. 2010
Sprague-Dawley rats	1 µg LPS (0.2 µg/µl )	16.5 dGA (term: 22 dGA)	PN 7	Alveolar simplification, as indicated by enlarged alveoli with decreased terminal airspace, decreased secondary septa and increased mean linear intercept	Li et al. 2016
Merino ewes	10 mg/sac LPS diluted in 50 µL NS	87, 92 dGA (prematurely delivered at 9 dGA, term: 147 dGA)	PN 0	Pulmonary arteriolar remodeling	Willems et al. 2017
Sprague-Dawley rats	10 µg ETX	20 dGA (term: 22 dGA)	PN 7, 14	Enlargement in parenchymal airspaces, a decrease in secondary septa, enlarged airspaces and decreased secondary septa	Mandell et al. 2021
Sprague–Dawley rats	10 µg/sac ETX^a^ diluted in 50 µL NS	20 dGA (term: 22 dGA)	PN 14	Sustained abnormalities of alveolar and vascular development, alters lung mechanics and causes right ventricular hypertrophy	Abele et al. 2022
Sprague–Dawley rats	10 µg/sac LPS diluted in 50 µL NS	20.5 dGA (term: 22 dGA)	PN 1, 7, 14	Alveolar simplification and reduced secondary septa	Zhou et al. 2022
Sprague–Dawley rats	*E. coli* suspension of 1MCF (2.5–3.5 × 10^8^ CFU/mL)	15 dGA (term: 22 dGA)	PN 3, 7, 14, 17, 19, 21	Inflammatory infiltration, impaired alveolar vesicular structure, less alveolar numbers, and thickened alveolar septa	Pan et al. 2023

^a^ dGA, days of gestational age; PN, postnatal days; ETX, endotoxin; NS, normal saline; LPS, lipopolysaccharides; *E. coli*, Escherichia coli

Morphometric analyses, reveal that prenatal LPS exposure in animals lead to fewer and larger alveoli with less secondary septa, enlarged distal airspaces and a reduced peripheral vessels density (Abele et al. 2022; Cao et al. 2009; Zhou et al. 2022). These changes in the alveoli suggest an arrest in alveolarization, but not destruction closely resembling “new” BPD (Li et al. 2016). Observed systemic maternal inflammation on postnatal alveolarization is transient, with catch-up alveolar growth later (Cao et al. 2009). Extremely premature ovine lungs exhibit exacerbated inflammation and vascular abnormalities when continuously exposed to bacteria and bacterial ETX in utero (Willems et al. 2017). A significant decrease in alveolarization and vascular development changed the lung structure to resemble BPD traits in humans (Abele et al. 2022; Li et al. 2016; Tang et al. 2010).

### Impacts of timing and duration of intrauterine inflammation exposure

Despite the potential link between intrauterine inflammation and BPD, multiple exposures to appropriate sequential intrauterine inflammation at varying times and durations may contribute to lung preconditioning and sensitization (Gussenhoven et al. 2017; Heymans et al. 2020; Kallapur et al. 2014). Studies indicate that intra-amniotic LPS exposure may shield premature lungs from additional inflammatory triggers by promoting immunological tolerance, reducing LPS-induced inhibition of alveolar and pulmonary vascular growth (Choi et al. 2016; Gisslen et al. 2014; Kunzmann et al. 2022; Kallapur et al. 2007; Kramer et al. 2005). Furthermore, a second intra-amniotic ETX injection suppressed monocyte function, including *E. coli* phagocytosis, hydrogen peroxide production, and IL-6 production (Kramer et al. 2005). Lambs repeatedly exposed to intra-amniotic LPS show lower expression of lung neutrophils, monocyte-inducible NO synthase, proinflammatory cytokine (IL-1β, IL-6, IL-8, and TNF-α), liver serum amyloid A3, TNF-α induced protein-3, etc. (Kunzmann et al. 2022; Kallapur et al. 2007). Moreover, lung remodelling similar to that observed in BPD occurred following single intra-amniotic LPS exposure. However, the inverse occurred after repeated intra-amniotic LPS exposure (Kallapur et al. 2005; Willet et al. 2000). These findings suggest that exposure to intra-amniotic LPS protects the lungs against BPD triggered by postnatal systemic inflammation.

Successive inflammatory exposure increases susceptibility to inflammatory responses, leading to exacerbation of the impact on pulmonary vascular development. In the study by Widowski et al. (2021), prenatal single inflammatory stimuli such as Ureaplasma or LPS inhibited the pro-angiogenic and vascular factors, including angiotensin (Ang)-1 and vascular endothelial growth factor (VEGF) receptor-2 (VEGFR-2). Previously exposure to Ureaplasma sensitized these indicators to an LPS-induced subsequent assault. Willems et al. (2017) demonstrated that chronic intra-amniotic Ureaplasma and subsequent LPS exposure promoted pulmonary inflammation, and reduced the expression of angiogenic growth factors and receptors compared to animals exposed to a single inflammatory event. In addition, a translational ovine model of intrauterine inflammation with successive exposure to Ureaplasma and LPS, negatively affected the epithelial stem/progenitor cell populations, resulting in reduced alveolar differentiation (Widowski et al. 2021).

## Mechanisms of intrauterine inflammation-related BPD

**Fig. 2:**
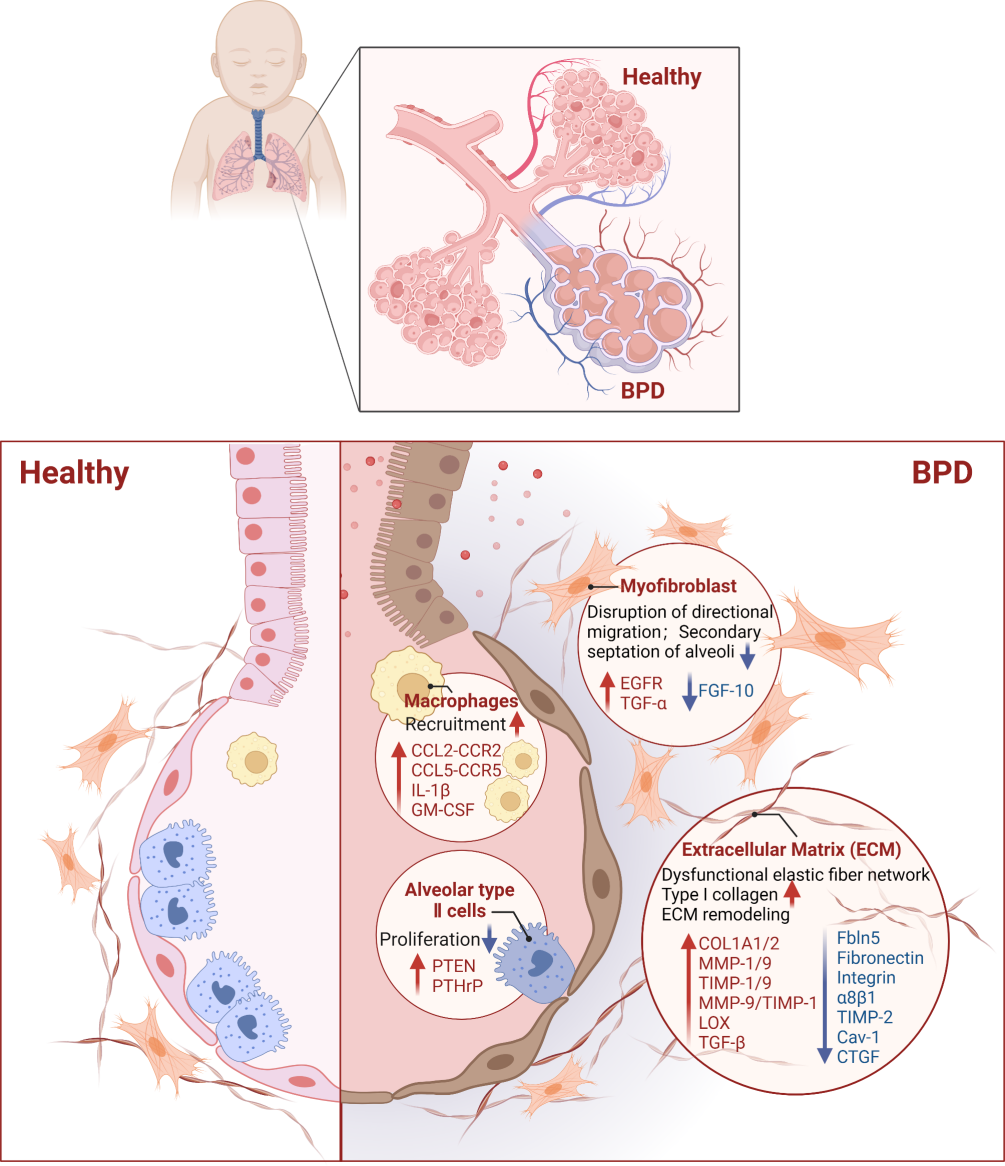
Potential mechanisms of intrauterine inflammation-related impaired lung alveolarization in BPD. The recruitment of macrophages, attenuated proliferation of ATII cells, abnormal directional migration of myofibroblasts, abnormal amount and crosslinking of extracellular matrix in the setting of intrauterine inflammation contribute to the arrested alveolar development in BPD, manifested by fewer and larger alveoli and fewer secondary septa. Created with Biorender. com

**Fig. 3:**
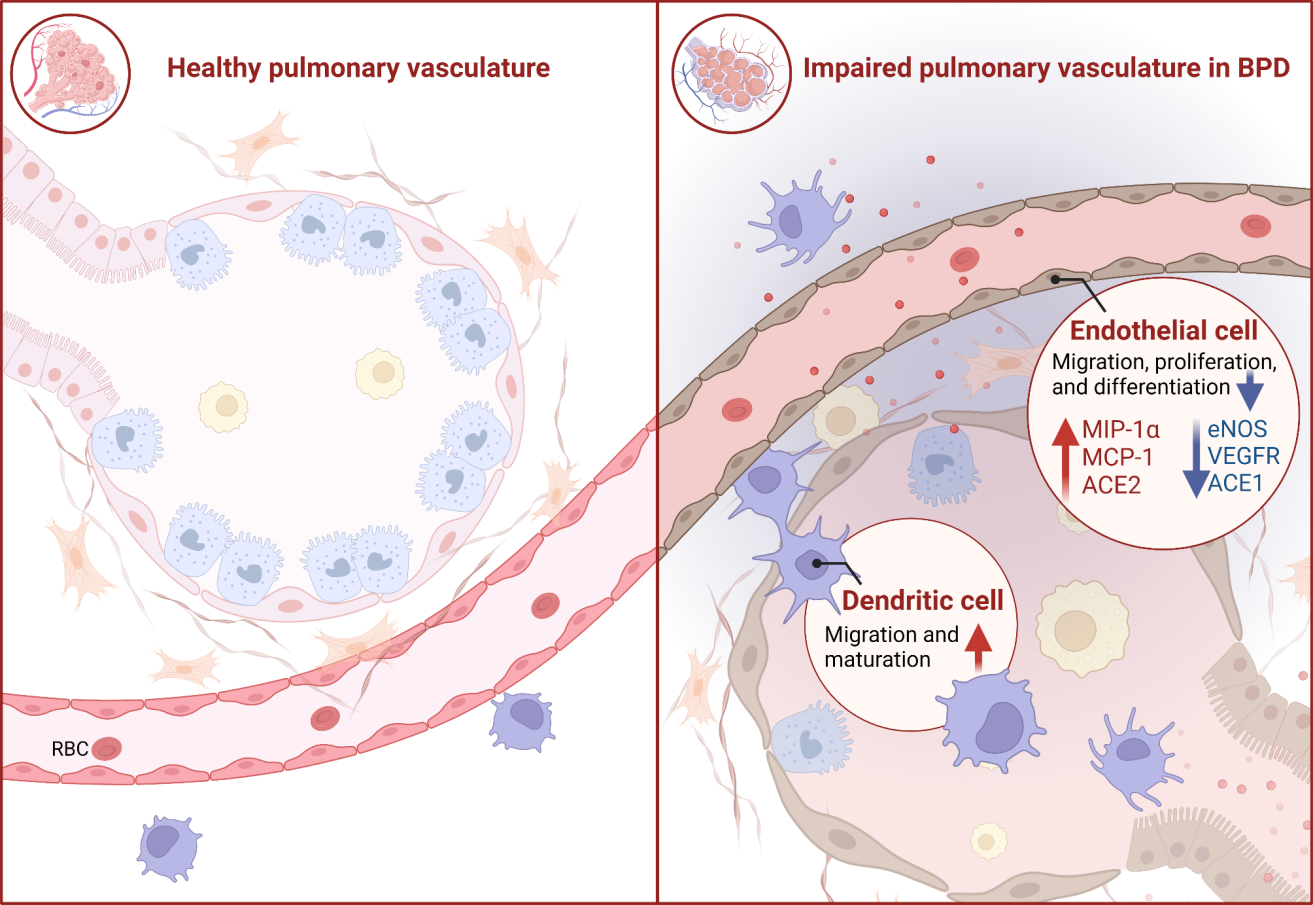
Potential mechanisms of intrauterine inflammation-related impaired pulmonary vascular development in BPD. Both the alteration of endothelial cell-related angiogenic factors and enzymes, and the migration and maturation of dendritic cells in the setting of intrauterine inflammation contribute to the disordered microvascular in BPD. Created with Biorender. com

We summarize the potential mechanisms by which intrauterine inflammation influences BPD etiology in terms of the impaired lung alveolarization (Fig. 2) and impaired pulmonary vascular development (Fig. 3).

## Impaired lung alveolarization

### Recruitment of macrophages and persistent lung inflammation

Some chemokines or cytokines produced by intrauterine inflammation exposure could lead to recruitment of macrophages in fetal lungs (Cui et al. 2020; Jackson et al. 2022). Key chemokine systems in macrophage recruitment, such as the C-C chemokine ligand 2 (CCL2)–C-C chemokine receptor 2 (CCR2) pathway and CCL5–CCR5 pathway are involved in BPD development. Repetitive LPS exposure induces CCL2–CCR2 expression, leading to exudative macrophages and contributing to hypoalveolarization (Cui et al. 2020). CCR5, predominantly expressed on macrophages, promotes immune cells migration, leading to undesirable inflammatory side effects (Costa et al. 2016). Blocking CCR5 in an intra-amniotic LPS-induced rat model decreases macrophage aggregation and enhances alveolarization, thereby ameliorating the BPD phenotype. Elevated CCR5 levels promote the production of IL-1β (Chen et al. 2021), which, secreted by activated fetal lung macrophages, is the principal inflammatory mediator disrupting airway morphogenesis and triggering alveolar hypoplasia in BPD (Stouch et al. 2016). Increased levels of granulocyte-macrophage colony-stimulating factor (GM-CSF), a pro-inflammatory cytokine, are present in chorioamnionitis-affected amniotic fluid or fetal lungs exposed to ETX. A surge in prenatal GM-CSF might stimulate an increase in the number of macrophages in the developing fetal lung. When subjected to a second exposure, such as hyperoxia, these accumulated interstitial macrophages are released into the alveolar space during postnatal life (Cheah et al. 2021b).

The recruitment of macrophages leads to a further imbalance between pro- and anti-inflammatory factors, which is another characteristic of BPD (Heydarian et al. 2022). Recent studies show that in BPD models, inflammation induced-classically activated/(M1) phenotype is aggravated, while alternatively activated/(M2) phenotype in macrophages is suppressed (Arora et al. 2018; Hirani et al. 2022; Syed and Bhandari 2013). Elevated macrophages levels produce neutrophil chemoattractant, inducing neutrophil migration out of pulmonary capillaries into the air space, resulting in continual lung inflammation (Kalymbetova et al. 2018; Xia et al. 2015). The inflammatory alveolar microenvironment reduces the survival of alveolar type II (ATII) cells while promoting matrix remodelling and activating fibroblasts (Hirani et al. 2022). Macrophages also contribute to pulmonary differentiation arrest by influencing the epithelial–mesenchymal transition process in ATII cells, likely being regulators of arrested alveolarization in BPD (Zhu et al. 2023).

### Attenuated proliferation of ATII cells

ATII cell populations decreased after intrauterine inflammation exposure, leading to impaired function restoration of alveolar epithelium morphology. In an intra-amniotic LPS injection model, LPS induces the phosphatase and tensin homolog, hindering ATK phosphorylation and ATII cell proliferation (Zhou et al. 2022). Normal lung growth and homeostasis depend on epithelial–mesenchymal interactions, which are mediated by the parathyroid hormone-related protein. LPS causes failure of the parathyroid hormone-related protein-driven epithelial-mesenchymal paracrine loop in the lung explants of rat fetuses. It therefore alters the effect of interstitial lipofibroblasts on ATII cells proliferation (Rehan et al. 2007). Furthermore, intra-amniotic LPS exposure in a lamb model resulted in a decrease in ATII cells after prenatal inflammation (Widowski et al. 2021). The ATII cells are stem cells responsible for homeostasis and regeneration in the alveolar compartments (Wu and Song 2020). Fiaturi et al. (2018) demonstrated the trans-differentiation of ATII cells into ATI cells. Once the number and function of ATII cells are compromised, ATII cells cannot rapidly proliferate and differentiate into ATI cells, compromising maintenance of alveolar homeostasis (Marega et al. 2023).

### Abnormal directional migration of myofibroblasts

Alveolar myofibroblasts, important for alveolar maturation, initiate a process called secondary septation during alveolar formation. Alveolar myofibroblasts are believed to migrate into the septal tips and lengthen secondary septa during alveolarization (Li and Gong 2020; Li et al. 2012). Intra-amniotic injection of LPS increases the migration speed, disrupts directional persistence, and causes an abnormal distribution of myofibroblasts in neonatal rat lungs. This leads to alveolarization arrest, which is a pathogenic mechanism of BPD (Li and Gong 2020; Li et al. 2012; Li et al. 2016). The actin cytoskeleton and cell migration in different cell types are significantly regulated by the epidermal growth factor receptor (EGFR) (Li et al. 2016). Intra-amniotic LPS exposure can activate EGFR by disrupting the asymmetric localization of phospho-EGFR (Li et al. 2012), leading to RhoA/Rho kinase activation and disruption of the directional migration of myofibroblasts (Li et al. 2016). In this process, EGFR phosphorylation is mediated by Src, which can be inhibited by the COOH-terminal Src kinase, suggesting that the COOH-terminal Src kinase/Src/EGFR signaling pathway may play a crucial role in modulating myofibroblast migration in a particular direction (Li et al. 2016). The 14-3-3*β* protein is also closely related to cytoskeletal remodelling and cell migration. LPS cannot activate RhoA or disturb myofibroblast polarization when 14-3-3*β* is knocked out, suggesting that 14-3-3*β* is required for RhoA activation by EGFR (Li and Gong 2020).

Transforming growth factor-α (TGF-α), a ligand of EGFR, has been proposed as the primary stimulus for maintaining myofibroblast polarity. This may contribute to arrested alveolar development in BPD. LPS treatment regulates EGFR activation by modulating TGF-α (Li and Gong 2020; Li et al. 2012). According to an earlier study, overexpression in the lungs disrupts postnatal alveolarization (Kramer et al. 2007). Moreover, intra-amniotic LPS exposure in a rat model led to the suppression of histone deacetylases 2 expression and activity, which induced TGF-α expression and disrupted alveolar morphology (Ni et al. 2014). Similar to TGF-α, LPS activated Src regulates cell surface metalloproteinase tumor necrosis factor-α converting enzyme (TACE) activity, leading to the cleavage of membrane-bound EGFR proligands, allowing them to attach to EGFR and activate downstream kinases (Li et al. 2016). Moreover, LPS-treated fetal mouse lung explants inhibit fibroblast growth factor-10 (FGF-10) expression by activating toll-like receptor 2 (TLR2) or TLR4.Consequently disrupted normal positioning of myofibroblasts around the saccular airways and abnormal saccular airway morphogenesis occurs (Benjamin et al. 2007).

### Abnormal amount and crosslinking of extracellular matrix (ECM)

The lung ECM consist of collagens, elastin, glycoproteins, and proteoglycans, providing structural scaffolding and biochemical support to tissues and cells (Liu et al. 2021; Mižíková and Morty 2015). Intrauterine inflammation may cause abnormal ECM composition, disorganized elastin and collagen fibers, and abnormal ECM crosslinking, contributing to arrested alveolarization in BPD (Mižíková et al. 2015; Rippa et al. 2021).

Elastic fiber formation in the respiratory region of the lungs is important for alveolar formation. Exposure to intrauterine inflammation disrupts elastic fiber organization and decreases elastin assembly components without redepositing elastin fibers or changing total lung elastin content (Benjamin et al. 2016; Hanita et al. 2017). Benjamin et al. (2016) showed that LPS could activate the NF-κB pathway through TLR4 while inhibiting the expression of Fbln5 in lung fibroblasts, resulting in dysfunctional elastic fiber network formation. Another study, using preterm lambs in an ovine model with intrauterine inflammation observed no tissue destruction, elastin fragmentation, or thick deposits of elastin. However, necrotizing funisitis resulted in a decreased volume density of the secondary septal crests. The same study suggested that the alveolar emphysema, a histological feature of “new” BPD, results from inhibited alveolarization (Hanita et al. 2017).

Collagens are highly abundant proteins in the ECM that provide resistance and support to tissues (Mižíková and Morty 2015). They have been shown to increase production in intrauterine inflammation-induced BPD animal models (Chou et al. 2016). Increased collagen area fraction was also observed in lung sections of patients with BPD (Ruschkowski et al. 2022). In a rat model of intrauterine inflammation, type I collagen expression significantly increased on embryonic day 21 and postnatal days 1, 3, 7, and 14, leading to abnormal lung development, with fewer alveoli and thickened alveolar septa in fetal and neonatal rats (Pan et al. 2018).

In the ECM, fibronectin, a high-molecular-weight glycoprotein, binds to cells through integrins that control migration and differentiation. Insufficient fibronectin levels may impede alveolar formation in premature lungs (Jin et al. 2020). In BPD, fibronectin, is suppressed and abnormally localized affecting airway proliferation and lung tissues (Benjamin et al. 2009; Jin et al. 2020; Prince et al. 2005). LPS actives NF-κB through functional TLR4, altering mesenchymal fibronectin expression. This interferes with interactions between epithelial and mesenchymal cells, as well as prevents distal airway branching and alveolarization (Prince et al. 2005). Jin et al. (2020) indicated that perinatal exposure to LPS in a mouse model of BPD caused the release of IL-33 from epithelial cells via myeloid differentiation factor 88, protein 38, and NF-κB protein 65. Elevated IL-33 in BPD triggered neutrophil extracellular trap formation via its receptor ST2, which degraded fibronectin in alveolar epithelial cells (Jin et al. 2020). LPS may also inhibit the fibronectin receptor integrin α8β1, resulting in a reduction of mesenchymal cell adhesion and migration (Benjamin et al. 2009).

Matrix metalloproteinases (MMPs), proteinases that participate in ECM degradation, are activated in inflamed tissue and inhibited by tissue inhibitors of matrix metalloproteinases (TIMPs) (Mižíková and Morty 2015; Zhou et al. 2018). Inflammatory responses mediated by IL-6, IL-1β, and tumor necrosis factor-α stimulate the secretion of MMPs (Goldenberg et al. 2008). The imbalance of MMPs and TIMPs may participate in the early inflammatory phase of BPD by regulating ECM remodelling. Yan et al. (2020) reported upregulated MMP-9 and TIMP-1 levels and MMP-9/TIMP-1 ratios in umbilical cord blood, considered predictive indicators of FIRS. MMP-9, TIMP-1, and MMP-9/TIMP-1 ratios in cord blood are closely correlated with the occurrence of BPD (Fukunaga et al. 2009). However, Lee et al. (2015a) reported that low TIMP-2 serum levels at birth may be associated with the subsequent development of BPD, and no significant differences were observed in MMP-8, MMP-9, or TIMP-1 levels. Therefore, different subsets of MMPs and TIMPs may play different roles in FIRS and BPD, which requires further evaluation.

TGF-β, a multifunctional polypeptide cytokine, is mainly secreted and stored in the ECM as a latent complex (Minton et al. 2014). TGF-β signaling activation in lung mesenchymal stromal cells enhance ECM deposition, inhibit branching morphogenesis and alveolarization, thereby promoting the progression of BPD (Aly et al. 2019; Chanda et al. 2019; Chen et al. 2011). Intra-amniotic ETX or LPS exposure increases lung TGF-β1 levels and induces the phosphorylation and translocation of the intracellular effectors of Smad2 to the nucleus (Collins et al. 2013; Kunzmann et al. 2007). Moreover, the expression of Cav-1, a structural component of caveolae that facilitates the degradation of TGF-β receptors, decreases after LPS exposure (Collins et al. 2013). The connective tissue growth factor (CTGF) is a downstream mediator of TGF-β1 that mediates the profibrotic effects of TGF-β1 by promoting fibroblast growth and ECM synthesis (Chen et al. 2011; Kunzmann et al. 2007). Increased CTGF levels contribute to fibrosis and pathogenesis of BPD after intra-amniotic LPS exposure (Collins et al. 2013). However, another study reported decreased concentration of CTGF in BPD led to microvasculature injury and alveologenesis, which is more consistent with the pathological changes associated with “new” BPD (Kunzmann et al. 2007).

## Impaired pulmonary vascular development

### Alteration of endothelial cell-related angiogenic factors and enzymes

Disordered microvascular development is another hallmark of BPD pathogenesis. The angiogenic factor VEGF is the most potent activator of angiogenesis and promotes the migration, proliferation, and differentiation of endothelial cells in existing vessels, thereby generating and maintaining new blood vessels (Lazarus and Keshet 2011; Laddha and Kulkarni 2019). Early VEGF-related intrauterine inflammation disrupts crucial growth factor signaling, which can lead to aberrant lung development and exacerbate BPD (Abman 2010). Damage during the pseudoglandular stage has been linked to markedly reduced VEGF expression in the lung tissue of fetal and neonatal rats (Pan et al. 2018). Decreased VEGFR expression has also been observed in a rat model of chorioamnionitis-induced BPD (Somashekar et al. 2016). Moreover, the treatment of newborn rats with a VEGFR inhibitor impairs pulmonary vascular growth and initiates pulmonary hypertension. These effects are long term and last well into adulthood (Le Cras et al. 2002). Besides the VEGF pathway, an early study showed that intra-amniotic LPS stimulates angiogenesis, migration, and proliferation in pulmonary cells, by inducing chemokines MIP-1α and MCP-1, leading to aberrant vascular development. The increased angiogenesis may expand the area between air spaces and capillaries and potentially creating a barrier to gas exchange, which is similar to aberrant alveolar capillaries in the BPD affected lungs (Miller et al. 2010).

Endothelial nitric oxide synthase (eNOS) crucial for angiogenesis and lung development, shows decreased activity in BPD models (Hirsch et al. 2020). Moreover, soluble endoglin is increased in the amniotic fluid of women with chorioamnionitis. Somashekar et al. (2016) showed that intrauterine exposure to an adenovirus overexpressing soluble endoglin decreased lung eNOS expression and phosphorylated Smad1/5 in rat pups. Simultaneously, a decrease in lung vascular density, increased pulmonary vascular remodelling, and increased medial wall thickness was observed. Angiotensin converting enzyme (ACE) is located mainly on the luminal surface of pulmonary endothelial cells and may be implicated in lung development. ACE is present in the human fetal lung at as early as 12 weeks of gestation, and its expression is downregulated in BPD lungs (Castro et al. 2014). ACE1 and ACE2 activities within the lungs counterbalance each other, with their ratio (ACE1/ACE2) correlating to the degree of lung injury (Wösten-van Asperen et al. 2011). Hillman et al. (2014) reported that exposure to intra-amniotic LPS or Ureaplasma parvum decreases ACE1 and increases ACE2, altering the ACE1/ACE2 ratio four-fold. This suggests that exposure to intrauterine inflammation-induced alterations in ACE may contribute to the development of BPD.

### Migration and maturation of dendritic cells

Dendritic cells, essential for normal postcanalicular lung development, may contribute to dysangiogenesis associated with BPD. Prenatal exposure to microorganisms may accelerate the migration and maturation of dendritic cells in the lungs of preterm infants. De Paepe et al. (2011) reported a more than three-fold increase in dendritic cells in the lungs of early control infants with a history of chorioamnionitis/prenatal infection. Thus, massive recruitment of dendritic cells to the lungs may be linked to the pathogenesis of BPD, possibly because of their ability to modulate angiogenesis (De Paepe et al. 2011).

## Prenatal treatment and prevention of intrauterine inflammation-related BPD

**Fig. 4:**
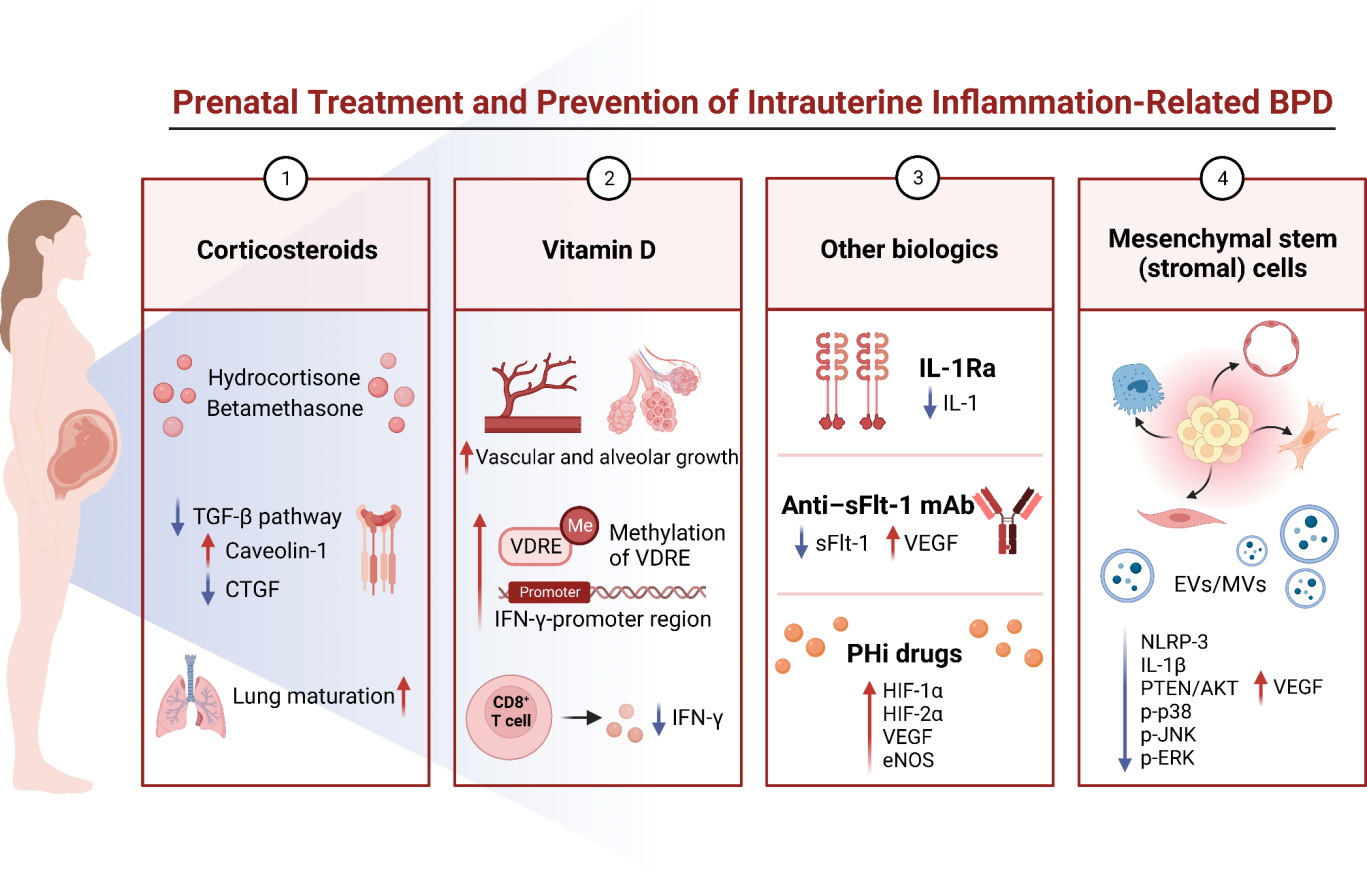
Prenatal treatment and prevention of intrauterine inflammation-related BPD. Corticosteroids, vitamin D, IL-1Ra, anti-sFlt-1 monoclonal antibody, prolyl-hydroxylase inhibitor drugs and mesenchymal stem (stromal) cells were proposed to be the prenatal treatment and prevention strategies based on the mechanisms of intrauterine inflammation-related BPD. Created with Biorender. com

As the important role of intrauterine inflammation in BPD became understood, several potential prenatal treatment and prevention strategies on BPD were proposed, keeping animal model evidence in consideration (Fig. 4). While some of these strategies have been clinical utilized, there is currently insufficient data to assess their effectiveness in addressing intrauterine inflammation-related BPD.

### Corticosteroids

Prenatal corticosteroids, a standard treatment for pregnancies at risk of preterm delivery between 24 and 34 weeks, have been used clinically for decades (Jobe and Goldenberg 2018). As potent anti-inflammatory agents, corticosteroids can suppress the inflammatory effects of intrauterine infections on the fetus, decreasing the risk of mortality and morbidity in preterm newborns (Conde-Agudelo et al. 2020; Ninan et al. 2022). A meta-analysis of seven observational studies suggested prenatal corticosteroids may effectively reduce adverse neonatal outcomes (e.g. neonatal mortality, respiratory distress syndrome, patent ductus arteriosus, intraventricular hemorrhage) caused by histological chorioamnionitis (Been et al. 2011).

While clinical research on the effects of prenatal corticosteroids in infants with BPD remain limited, animal models suggest great potential impact. Those studies have shown that LPS and corticosteroid-mediated molecules interact in the developing lungs, affecting the prenatal LPS-induced intrauterine inflammation model. In an ovine intrauterine inflammation model, maternal intramuscular injection of betamethasone (0.5 mg/kg) before delivery counteracted LPS-induced TGF-β pathway activation. Moreover, caveolin-1, which enhances TGF-β receptor degradation, was increased. Betamethasone treatment seven days before exposure to LPS prevented an increase in the downstream TGF-β signaling CTGF levels (Collins et al. 2013). Significantly, the effect of prenatal corticosteroids on intrauterine inflammation-related BPD varies according to the timing of administration. Kuypers et al. (2012) indicated that lung inflammation is modulated by the exposure time to corticosteroids, which are related to pro-inflammatory stimuli. Their study demonstrated that betamethasone treatment administered seven days after intra-amniotic LPS failed to diminish lung inflammation and instead accelerated lung maturation. However, lung inflammation was relieved and lung maturation was more restrained when betamethasone was applied seven days prior to intra-amniotic LPS (Kuypers et al. 2012).

The use of corticosteroids is controversial due to their immunosuppressive effects, which may raise the risk of neonatal infection and/or exacerbate systemic infections of mother (Conde-Agudelo et al. 2020). However, the results of the meta-analysis of the effects of prenatal corticosteroids in preterm labor, with clinical or histological chorioamnionitis showed that there was no significantly increased risk (Been et al. 2011). Another Cochrane review showed that, for the pregnant women, prenatal corticosteroids therapy does not raise the risk of endometritis, puerperal sepsis, or chorioamnionitis (Roberts et al. 2017). In brief, randomized controlled trials evaluating the efficacy of prenatal corticosteroids on preventing intrauterine inflammation-related BPD are relatively safe and expected.

### Vitamin D

Vitamin D is a sterol hormone with anti-inflammatory properties that modulates normal fetal lung development (Mandell et al. 2015). Vitamin D deficiency has been reported in 70% of preterm infants (Park et al. 2020). According to a meta-analysis that included 909 infants, prenatal vitamin D deficiency and low vitamin D levels are associated with neonatal BPD (Park et al. 2020). Early vitamin D supplementation could thus significantly reduce inflammatory response thereby preventing and reducing BPD incidence in preterm infants (Ge et al. 2022). Mandell et al. (2014) reported that intra-amniotic vitamin D injection treatment at 14 days in fetal rats improved alveolar and vascular growth by 45% and 25%, respectively. In addition, vitamin D enhanced fetal ATII cell growth in the presence of ETX-induced growth suppression by 73%, suggesting that the effect of prenatal vitamin D therapy may be partially explained by its direct influence on alveolar and vessel growth (Mandell et al. 2014). Another study demonstrated that vitamin D administration alleviates prenatal inflammatory damage by inhibiting the synthesis of IFN-γ. Liu et al. (2018) discovered that infants whose mothers had mid-pregnancy vitamin D concentrations of ≥ 30 ng/mL had considerably lower levels of IFN-γ. It is therefore reasonable to infer that IFN-γ may be a key factor in the involvement of vitamin D deficiency in intrauterine inflammation-related BPD. Specifically, vitamin D boosts the methylation of vitamin D response elements in the IFN-γ-promoter region and inhibits the LPS-induced expression of IFN-γ production in CD8^+^ T cells, leading to enhanced maturation of alveoli in an intra-amniotic LPS-induced BPD rat model (Liu et al. 2018). The effect of prenatal vitamin D supplementation on intrauterine inflammation-related BPD is promising, however, more mechanism studies and clinical trial evidence are needed.

### Other biologics: IL-1Ra, anti-sflt-1 monoclonal antibody, and prolyl-hydroxylase inhibitor drugs

IL-1 is a central inflammatory cytokine present in the chorioamniotic fluid and lung effluent of preterm infants after birth (Stouch et al. 2016). Studies have demonstrated that prophylactic administration of an IL-1 receptor antagonist (IL-1Ra) almost completely protects against BPD and permits almost normal lung development in mouse models with clinical relevance (Nold et al. 2013). A similar observation in an ovine model proved that IL-1Ra reduced fetal pulmonary and systemic inflammatory responses to intra-amniotic LPS (Kallapur et al. 2009). While the therapeutic effect of IL-1Ra on BPD remain unproven, the favorable safety profile of IL-1Ra is well established by a cohort study. Including 26 patients ages 0.80–42.17 years who were treated with IL-1Ra for at least 36 months, this study created a substantial track record for the safety of this treatment (Sibley et al. 2012). Soluble fms-like tyrosine kinase1 (sFlt-1) is an endogenous antagonist of VEGF that inhibits VEGF activity by directly interacting with free VEGF protein. These results in decreased downstream activation of membrane-associated VEGF receptors. Significantly, prenatal anti-sFlt-1 monoclonal antibody therapy preserved lung alveolar and vascular growth in an intrauterine inflammation model of BPD. It also reduced the adverse lung effects of intra-amniotic ETX (Wallace et al. 2018). Hypoxia-inducible factors (HIF), which are potent upstream regulators of diverse signaling pathways, also play an important role in supporting normal lung development (Semenza 2019). HIF signaling can be strengthened through pharmacological therapy with prolyl-hydroxylase inhibitor drugs that stabilize HIF. Prenatal selective prolyl-hydroxylase inhibitor drugs, such as dimethyloxalylglycine or GSK360A, increase the lung protein content of both HIF isomers, HIF-1α and HIF-2α, as well as VEGF and eNOS. They also preserve lung growth and function following prenatal ETX exposure, thereby preventing pulmonary hypertension in a rat model of chorioamnionitis-induced BPD (Hirsch et al. 2020). However, the clinical application of anti-sFlt-1 monoclonal antibody and prolyl-hydroxylase inhibitors, especially for pregnant women in the late stages of pregnancy, still requires more evaluation.

### Mesenchymal stem (stromal) cells

Mesenchymal stem (stromal) cells, originating from embryonic splanchnic mesoderm, shows their importance by differentiating into various lung cells (Zhang et al. 2023b). It can coordinate key processes in lung morphogenesis by differentiating into either alveolar and vascular cells, or into fibroblasts and smooth muscle cells, which are crucial in the pathogenesis of BPD (Aly et al. 2019). The preventive effect of mesenchymal stem (stromal) cells against BPD in animal models appears to be mediated by secreted extracellular vesicles (Abele et al. 2022). Significantly, prenatal extracellular vesicle treatment significantly reduced the expression of inflammasome gene signals NLRP-3 and IL-1β in a chorioamnionitis-induced BPD model, whilst enhancing distal lung branching. It also preserved normal lung angiogenesis and vascular growth by increasing VEGF expression (Abele et al. 2022). Specifically, microvesicles, a type of extracellular vesicle that originate from cell membranes, enhance lung morphology and development, reduce lung fibrosis, repair peripheral pulmonary blood vessel loss, and mitigate pulmonary vascular remodelling. This assists normal lung development and their action is possibly associated with modulation of lung macrophage phenotype (Willis et al. 2018). In an intrauterine LPS-induced BPD model, microvesicle treatment on postnatal day seven improved alveolarization by promoting the proliferation of AT2 cells, which may be mediated by inhibition of the phosphatase and tensin homolog/AKT pathway. Microvesicle treatment also attenuates lung inflammation caused by prenatal LPS exposure by suppressing phospho-protein 38, phospho-JNK, and phospho-ERK expression (Zhou et al. 2022). Therefore, extracellular vesicle treatment may be a novel therapeutic strategy for the prevention of BPD caused by prenatal inflammation. Clinical trials show the promise of mesenchymal stem (stromal) cells therapy in preventing and treating BPD in early preterm infants (Yilmaz et al. 2021; Wu et al. 2020). Further clinical trials are required to demonstrate the efficacy and safety of prenatal mesenchymal stem (stromal) cells therapy for BPD, even if preclinical studies have confirmed its significance.

## Conclusion

In summary, targeting inflammation within the womb is the critical step in preventing bronchopulmonary dysplasia, a condition that disrupts healthy lung development in premature infants. Several promising prenatal therapeutic strategies have been introduced, yet future challenges include identifying ways to better predict BPD based on evidence of prenatal inflammation and developing innovative and preventive management strategies for BPD.

## Data Availability

Not applicable.

## Abbreviations

ACE: Angiotensin converting enzyme
ATI: Alveolar type I
ATII: Alveolar type II
BPD: Bronchopulmonary dysplasia
CCL2: C-C chemokine ligand 2
CCR2: C-C chemokine receptor 2
CTGF: Connective tissue growth factor
ECM: Extracellular matrix
EGFR: Epidermal growth factor receptor
eNOS: Endothelial nitric oxide synthase
ETX: Endotoxin
FGF-10: Fibroblast growth factor-10
FIR: Fetal inflammatory response
FIRS: Fetal inflammatory response syndrome
GM-CSF: Granulocyte-macrophage colony-stimulating factor
HIF: Hypoxia-inducible factors
IL-1Ra: IL-1 receptor antagonist
IL-6: Interleukin 6
LPS: Lipopolysaccharides
MIR: Maternal inflammatory response
MMPs: Matrix metalloproteinases
MV: Mechanical ventilation
sFlt-1: Soluble fms-like tyrosine kinase1
TACE: Tumor necrosis factor-α converting enzyme
TGF-α: Transforming growth factor-α
TIMPs: Tissue inhibitors of matrix metalloproteinases
TLR2: Toll-like receptor 2
VEGF: Vascular endothelial growth factor
VEGFR-2: Vascular endothelial growth factor receptor-2
